# Facial Motion Engages Predictive Visual Mechanisms

**DOI:** 10.1371/journal.pone.0091038

**Published:** 2014-03-14

**Authors:** Jordy Kaufman, Patrick J. Johnston

**Affiliations:** 1 Swinburne University of Technology, Hawthorn, Victoria, Australia; 2 Department of Psychology, University of York, York, United Kingdom; University of British Columbia, Canada

## Abstract

We employed a novel cuing paradigm to assess whether dynamically versus statically presented facial expressions differentially engaged predictive visual mechanisms. Participants were presented with a cueing stimulus that was either the static depiction of a low intensity expressed emotion; or a dynamic sequence evolving from a neutral expression to the low intensity expressed emotion. Following this cue and a backwards mask, participants were presented with a probe face that displayed either the same emotion (congruent) or a different emotion (incongruent) with respect to that displayed by the cue although expressed at a high intensity. The probe face had either the same or different identity from the cued face. The participants' task was to indicate whether or not the probe face showed the same emotion as the cue. Dynamic cues and same identity cues both led to a greater tendency towards congruent responding, although these factors did not interact. Facial motion also led to faster responding when the probe face was emotionally congruent to the cue. We interpret these results as indicating that dynamic facial displays preferentially invoke predictive visual mechanisms, and suggest that motoric simulation may provide an important basis for the generation of predictions in the visual system.

## Introduction

The ability to make rapid judgements about the emotional states of conspecifics from their facial displays is a fundamental component of the human neurocognitive system [1]. Despite much research into facial affect processing, historically the use of static (non-moving) face stimuli has been the norm, and it is only relatively recently that the use of dynamic (moving) stimulus materials has become more commonly used [2]–[5]. The historic reliance on non-moving stimuli is counterintuitive as real-life facial expressions are dynamic, and correspondingly dynamic stimulus materials should promote ecological validity. Moreover, facial expressions of affect are often explicitly characterised in terms of dynamic actions (e.g., [6]), and the predominant neurocognitive models of face processing [7] emphasise separable processing mechanisms for dynamic aspects of faces. That the preponderance of studies conducted in the area has used static pictures of facial affect may, in part, reflect difficulties in achieving adequately controlled stimuli. It also reflects historical limitations in terms of stimulus delivery systems (which recent work suggests may be surmountable [8]).

Where dynamic stimuli have been used, indications are that they may facilitate facial affect processing [9]. Some research reports dynamic displays of emotion being more easily recognised than static displays [9], [10]; although other studies fail to report this [2], [3]. Further studies suggest that dynamic facial affect displays lead to greater arousal than static displays [11]; and elicit more spontaneous mimicry [12]. Dynamic face stimuli may have broader facilitatory effects than those observed in affective processing. Age processing [13] and identity processing [14] may be improved to dynamic stimuli, thus implying that our neurocognitive system is highly effective at extracting subtle cues from facial actions.

Recent neuroscientific investigations have also examined the effects of dynamic stimuli, and suggest that moving facial expressions invoke greater activation in posterior brain regions, particularly MT/V5+ and posterior Superior Temporal Sulcus with some studies also showing greater activation in the fusiform gyrus [4], [15]–[18]. Neuroimaging studies of facial affect processing have also regularly reported activation in inferior parietal and inferior frontal brain regions as well as the supplementary motor area [4], [18]–[22] leading to the suggestion that the Mirror Neuron System (MNS) might play a role in facial affect recognition. Mirror neurons in the premotor and inferior parietal cortices, active during both the execution of action and the observation of that same action [23], have been proposed to be involved in modelling, imitating and understanding of behaviour [24].

A number of studies offer support for a role for the MNS in facial affect processing; dynamically presented facial emotions invoke spontaneous mimicry [25], and a state-dependent transcranial magnetic stimulation study [26] has shown that behavioural accuracy in face emotion recognition is correlated with an index of MNS efficacy. Whilst, the idea that the MNS underpins high-level understanding of the intentions and mental states remains controversial [27], it is now well established that viewing the meaningful motor behaviours of other humans leads to activation of a network of brain structures that can broadly be considered to play a role in functions relating to action-observation, action-planning, mimicry and motor imagery, and to emotional evaluation and empathy [18], [19].

One suggestion that attempts to resolve the role of motor representations in action understanding has been the “predictive coding” framework [28] based on Von Helmholtz' notion of “unconscious inference.” [29]. Predictive coding is suggested to be a general property of the neurocognitive system, the central idea of which is that rather than simply passively registering sensory data, the brain actively predicts what its sensory input will be on an ongoing basis. By adopting such a strategy the brain is hypothesized to minimize the computational burden placed upon it in deciphering sensory inputs [30], and thereby implement an energy efficient solution to the problem. Predictive coding models postulate that the final percept is derived through the resolution of mutual information or minimization of error within a cascaded network of reciprocally interconnected systems (e.g., [31]). In the context of the visual perception of action, the suggestion is that reciprocal feedforward-feedback loops between visual areas and motor areas instantiate a system for prediction generation and error-checking with respect to the actual visual input relating to observed motor acts. The theoretical plausibility of this notion has been demonstrated though neural network simulation studies [32].

Tentative behavioural evidence for predictive coding mechanisms in relation to the brain's response to facial emotion stimuli comes from a study by Yoshikawa and Sato [5], who reported that dynamic facial emotion stimuli induce “representational momentum”. That is, the final frame of a facial motion sequence was evaluated as having shown greater emotional intensity than was actually displayed. The extent of representational momentum was partially dependent on stimulus velocity. Taken together, these results imply an internal modelling/prediction of the stimulus trajectory rather than simple pattern recognition. In the context of the existing literature on mimicry and the involvement of MNS structures in facial emotion processing, the actions of mirror neurons provide a plausible basis for such trajectory modelling. It is proposed that such modelling may facilitate expression recognition by generating predictions.

The current study aimed to explore the potential functional consequences of the predictive mechanisms implied by representational momentum phenomena with respect to facial affect recognition. We employed a novel cueing paradigm whereby participants were cued by either the static or dynamic presentation of a non-apical intensity emotion and subsequently presented with a probe showing the same actor either expressing the full intensity of either the cued emotion, or a different emotion. The participants' task was to judge whether the probe stimulus was congruent (same emotion) or incongruent (different emotion) to the cueing stimulus. We hypothesised that dynamically presented facial expression stimuli would preferentially engage motor simulation mechanisms, and that such engagement would bias expectation with respect to the following probe stimulus. We therefore predicted an effect of stimulus motion such that dynamic stimuli would lead participants to form an implicit expectation of stimulus congruence. Thus, when cued with dynamic stimuli, participants would be fastest and most accurate in congruent trials and slowest and least accurate with incongruent trials. In other words, dynamic stimuli will induce representational momentum such that participants are biased towards making “congruent” responses.

Additionally, we added a same/different identity manipulation to this experiment. This was important aspect of our study because it allows this work to overcome a potentially difficult confound regarding emotion processing vs. trajectory processing. Without this condition, it would be possible to argue that any predictive mechanisms in play are not necessarily working with motoric input, but instead could be based on simple ballistic trajectories of particular facial elements (e.g., the corners of the mouth during a partial smile), such that participants react to a specific facial feature is or is not where it should be depending on whether the target is congruent or incongruent.

Although existing literature on facial affect processing raises the likelihood of differential effects across different emotions (e.g., [33]) specific predictions in this regard were beyond the scope of the current study. The existing literature on differential performance across emotion categories as a function of stimulus dynamism is inconsistent, and the theoretical grounding from which to make specific predictions in this regard is not yet clearly established.

## Methods

### Participants

Twenty-one adults (16 female, 21 white Caucasian) gave informed consent and participated in the study. Participants were university students ranged 19 to 28 years (M = 21.9, SD = 3.2) with no history of neurological/psychiatric illness. All participants provided written consent prior to participating in this study. The consent instruments and the experimental procedures were approved by the Department of Psychology Ethics Committee at University of York.

### Stimuli

Stimuli were derived from the NIMSTIM set (MacArthur Foundation Research Network on Early Experience and Brain Development; http://www.macbrain.org/resources.htm). Following Mayes et al. [2], Abrosoft FantaMorph was used to create the dynamic stimuli from pairs of static images, (neutral and emotional poses for the same actor). For each image pair, a minimum of 45 corresponding spatial points was co-identified (key locations including, inner and outer canthi of eyes, pupil centres and locations along the top and bottom of the upper and lower lip). Using these matches, morphs of 30 physically equal steps were created, producing 30 sequential images (one-second video-clips at 30fps) showing a neutral repose evolve into a fully expressed emotion. Twelve dynamic stimuli, comprising exemplars each of three emotions (happiness, anger, and fear; two Caucasian male, one Caucasian female and one Asian female poser) were created. There were an equal number of static stimuli (same posers/emotions) – for both 100% (i.e., the full apical extent of expression) and 50% expressions (i.e. the morph's physical mid-point). In a piloting exercise, 51 participants (40 female; M = 21.3 years, SD = 3.3) made 5-alternative-forced-choice emotion category judgements on these stimuli along with two other emotions: disgust and sadness. Recognition accuracy of the stimulus categories was as follows: happiness at 94%, fear at 68% and anger at 67%.

### Procedure

Trials involved two phases; a cueing phase followed by a probe phase. For each trial the cueing stimulus was either dynamic or static (randomly assigned across trials). In the *dynamic condition*, an emotionally neutral face appeared which evolved dynamically over the period of 500 ms to the non-apical expression of an emotion (i.e. 50% of full intensity). The face was then masked by a cross-hatch pattern presented for 500 ms. The cueing phase of the *static condition* was the same except that instead of presenting a dynamically changing face, only the non-apical emotional face appeared (for 500 ms), prior to the appearance of the cross-hatch mask. The probe phase was identical for both the *static* and *dynamic* conditions.

**Figure 1:**
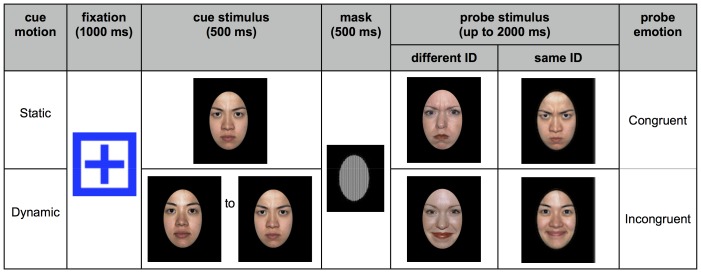
Experimental trial structure and stimulus examples. The faces, displayed in this figure were obtained from the NimStim facial stimulus set [40]. Consent to use these specific images was granted for publication purposes by the NimStim stimulus set developers (http://www.macbrain.org/resources.htm).

The probe stimulus was always static and was always had 100% emotional intensity. This face expressed either the same emotion as the face shown in the cuing phase (*congruent*) or a different fully expressed emotion (*incongruent*). For half the trials the same actor appeared in both phases (*same identity condition*), and for the other half two different (but same-gendered) actors were used (*different identity condition*). The participant's task was to indicate (via button press) whether the probe emotion was the same (*congruent*) or different (*incongruent*) with the (partial intensity) emotion of the cueing phase (regardless of actor identity). The probe remained on the screen until a response was made. Failures to respond within two seconds were coded as incorrect. Trial structure is displayed schematically in Figure 1.

Each of the six specific incongruent emotion stimulus pairing occurred with equal likelihood. Each of the three congruent pairings occurred with a greater likelihood to help match the conditions in frequency. In total 240 trials were presented to each participant which varied in emotion of the prime, emotional congruence of the probe with the prime, identity of the actor, gender of the actor (always consistent from prime phase to probe phase), and type of motion (static or dynamic). Stimuli were presented and responses were logged using the software package *Presentation*. Participants used different hands for congruent/incongruent responses (counterbalanced across participants). Participants sat 70 cm from a 34×21.2 cm LCD monitor. All visual stimuli subtended a horizontal visual angle of 5.0 degrees and vertical visual angle of 7.7 degrees.

All bias, accuracy and reaction time data were analysed using the statistical software package, JMP.

## Results

### Bias

**Figure 2:**
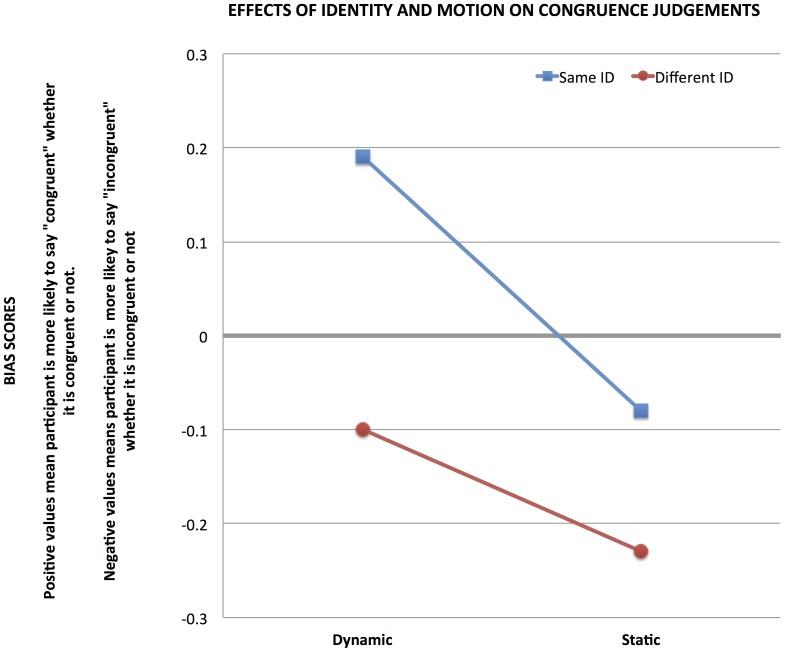
Dynamic facial motion by the cued face biased participants to indicate that the probe face was congruent. Maintaining the same identity from cue to target also biased participants in this way.

To measure the extent to which different priming types biased respondents towards making a congruent response, we calculated the bias statistic: *c* [34]. This statistic is calculated as: 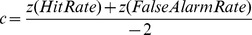



In this case, hit rates refer to a correct response of “congruent” while false alarms refer to an incorrect response of “congruent”. The c statistic is zero when there is no bias. Negative c scores denote a bias towards making a “congruent” response whereas positive c scores demonstrate a bias towards responding “incongruent.” A repeated-measures 2 X 2 ANOVA (identity X motion) revealed a main effect for stimulus motion F(1,20) = 14.24, p = .0012, such that dynamic primes increased participant bias (c = .04) towards indicating the probe was congruent relative to the static cue (c = −.16). Face identity also had a significant effect on participant bias, with same identity faces lending to significantly greater congruence bias (c = .05) than different identity faces (c = −.17). A significant interaction was not observed for identity by motion, F(1,20) = 2.8, p = .11. The bias data is plotted in Figure 2. The bias components (hit rate and false alarms) are detailed in Table 1. In summary, motion in the cue stimulus biased participants towards congruent responding, as did the sharing of person identity between the cue stimulus and the probe stimulus, although these factors did not interact.

**Table 1:** Hit and False Alarm rates by identity and motion conditions.

Identity	Motion	Hits		False Alarms	
		%	SD	%	SD
Same	Static	74%	.08	22%	.1
Same	Dynamic	83%	.09	28%	.13
Different	Static	65%	.15	21%	.12
Different	Dynamic	74%	.14	20%	.11

### Reaction Times

**Figure 3:**
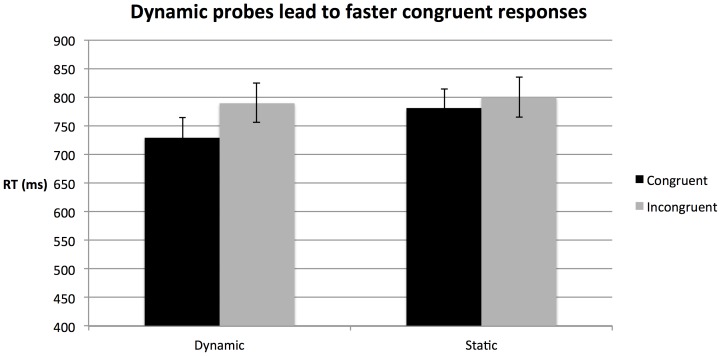
Dynamic facial motion by the cued face led participants to more quickly correctly identify the probe face as “congruent” than as “incongruent.” Static facial cues did not have this effect.

To assess our hypothesis that dynamic stimuli prepare participants for a congruent emotion, analyses focussed on the difference in response speed to congruent and incongruent stimuli: RT(difference)  =  RT(incongruent) – RT(congruent); thus negative values indicate faster responding to the congruent emotion. A repeated measures ANOVA (motion X identity) for RT(difference) revealed a main effect of motion, F(1,20) = 6.48, p = 0.0192, indicating that compared with static cues, dynamic cues led participants to respond more quickly to congruent stimuli relative to incongruent stimuli. Comparing RT(difference) score to zero (i.e., zero indicates no speed advantage to incongruent or congruent stimuli), determined that dynamic primes led to significantly faster responding to congruent stimuli than to incongruent stimuli, t(20) = −5.14, p<0.001. For static primes, there was no significant advantage to congruent or incongruent stimuli, t(20) = −1.69, p = .1070. There was no significant effect of identity, F(1,20) = 2.57, p = .1244, nor was there a significant motion by identity interaction, F(1,20) = .003, p = .9594. The reaction time data is plotted in Figure 3. To summarise, dynamic but not static cues led to a speed advantage for responding to emotionally congruent probe stimuli.

## Discussion

The fundamental contribution of this paper is the finding that human mind will process very brief and subtle emotional face presentations, provided that such presentations are dynamic in nature; and that this has practical effects in subsequent face processing. More specifically our results indicate that dynamically changing emotional stimuli set in motion processes leading the viewer to expect to see the congruent end point of this brief and non-apical presentation. As such, it appears that like in other areas of visual cognition, predictive visual mechanisms have an important role to play in emotion processing.

This conclusion is based on analysis of both responses accuracy and reaction time in this novel dynamic cueing experiment. With respect to accuracy, our key measure was participant bias to indicate that the target face was congruent with the cue. Analysis of bias (which is a function of both hit and false alarm rate) demonstrated that dynamic presentations led participants to say that the final face was congruent–whether it was so or not. Thus, for example, watching a face change from a neutral expression to a partially fearful expression, made the participant more likely to indicate that the target face was fearful–generating a “hit” when the target face was in fact fearful; and/or a false alarm when the face was happy, sad or angry. We propose that the most parsimonious explanation of this finding is that stimulus motion in the cue has engaged *predictive simulational* mechanisms that have generated an *expectancy bias* with respect to the emotional expression of the probe stimulus.

Evidence of such an *expectancy bias* with respect to the probe stimulus is also seen in analysis of reaction times. Here, we find that dynamic cueing led participants to respond significantly faster when the target face was congruent than when it was incongruent. In contrast, static cueing did not lead to a difference in response speed as a function of target congruence. This is strong evidence for *predictive simulation* or emotional representational momentum because, by this account, the predictive mechanism based on this pre-processing should have participants form a representation of an emotionally congruent target face.

It is noted that our experiment, in some respects, resembles well-documented affective *congruence priming* phenomena, whereby, pre-exposure to a particularly valenced stimuli facilitates the subsequent responding to similarly valenced material [35]. Importantly, the current paradigm differs significantly from the typical congruence priming situation, since, generally, there is no dependency between the cue/prime stimulus and the task demands to the target stimulus, whereas in the current study the appropriate response to the probe/target stimulus is determined with explicit reference to the cue. Although the current experiment shares some superficial similarities to congruence priming phenomena, we do not believe that explanatory frameworks offered by that literature are able to account for our current results. In general, explanations of affective priming phenomena have focussed upon either processes operating at the stimulus encoding by pre-activating relevant memory traces [36] or at the response selection by pre-activating response tendencies [37]. However, the effect that we report is not a typical congruence priming effect, since we do not report a general facilitated responding to emotionally congruent pairings, but rather, a bias towards “congruent” responding that is *specific to dynamic cues*. That is to say, our dependent variable indexes the *expectancy of congruence* rather than facilitatory effects of congruent pairings. That this expectancy is subject to the influence of stimulus motion lies outside of the purview of theories of congruence priming, and is more consistent with the predictive simulation model that we have proposed.

The finding of representational momentum with respect to emotional processing fits well with recent work by Jellema and colleagues [38]. In this study participants were presented with similar stimuli as the faces were shown to morph from a 100% emotion (happy or angry) to neutral, whereupon the participants had to indicate how they perceived the emotion of the face in the final frame. When the face began happy and ended neutral, participants viewed the final frame as being slightly angry; and they viewed the final angry-to-neutral sequence frame as being slightly happy. This appears to occur because, as with our results, the dynamically presented stimuli elicit representational momentum in emotional processing.

While the key finding of this work is based on analyses of dynamic vs. static cue emotion, the results of manipulating facial identify are also worthy of discussion. A reoccurring finding in this work was the strong effect of facial identity on participant accuracy. When the face retained its identity from the cueing phase to the target phase, respondents were more likely to indicate that the target was congruent. The augmented bias towards an “incongruent” response in the different-identity condition, may partially stem may from the the fact that in the different-identity condition there was an increased likelihood of a cueing or probe face being presented that was ethnically incongruent with the participant. Since within the different-identity condition all of the trials involving female actors involved a concurrent change of race with the change of identity (i.e. there were two actors who were ethnically different) the identity-change condition is partially confounded, and any effects could be due to either changes in identity or changes in race. Importantly, however there was no clear interaction of cue motion with facial identity. In other words, our results show that the predictive mechanisms set in motion by dynamic cues can be argued to relate to emotion processing independently from the processing of facial identity. As such, a mechanism based on trajectory mapping of specific facial features is not supported by our data. Our data is more consistent with predictions based upon embodied motoric simulation biasing expectations.

It is worth noting that our stimuli only used 50% apical emotions for static and dynamic cues. This leaves the door open to future research to further investigate if there is a minimum level of dynamic motion necessary or static presentation necessary to elicit this predictive mechanism. Also, although facial stimuli morphs like those used here are common in the emotion processing literature, more ecologically valid stimuli (including seeing actual faces) which entail onset latencies that vary with each facial feature [39] may more dramatically reveal the predictive mechanism shown to be at play here. Also, as discussed above, it is worth considering the potential influence of ethnicity on our results. We did not analyze our data with respect to race and racial congruence between the participant and the stimuli used could potentially interact with our reported effects. Future research will undoubtedly clarify each of these issues.

Another interesting direction for future research is an investigation into the nature of the dynamic motion necessary to elicit the predictive coding found here. It has been our assumption that dynamic facial motion led to the bias towards congruence. Although this may appear to be the most obvious explanation for our results, it is hypothetically possible that any dynamic motion prior to the presentation of the probe stimulus could have elicited this bias (e.g., a moving face with an unchanging non-apical facial expression, or even a dynamically moving background). Although, unlikely in our view, this is a possibility worth examining and the results would better inform our understanding of the predicative mechanisms underlying face processing.

In conclusion, this report introduces a novel cueing paradigm that demonstrates that dynamic facial displays bias viewer expectations. We interpret this as indicating that facial motion invokes predictive simulational mechanisms that may guide visual perception and have functional consequences for facial affect recognition. Future work will adapt this new cueing paradigm for use in neuroimaging investigation of emotional face processing as it provides an approach to minimising potentially serious confounds inherent in comparing brain responses to dynamic and static emotional faces, and for addressing fundamental questions relating to the localisation and timing of prediction and error-checking mechanisms involved in visual perception.
